# SUGAMMADEX versus neostigmine after ROCURONIUM continuous infusion in patients undergoing liver transplantation

**DOI:** 10.1186/s12871-020-00986-z

**Published:** 2020-03-25

**Authors:** Cristian Deana, Federico Barbariol, Stefano D’Incà, Livia Pompei, Giorgio Della Rocca

**Affiliations:** 1 Anesthesia and Intensive Care 1, Department of Anesthesia and Intensive Care Medicine, Academic Hospital “S. Maria della Misericordia”, Piazzale S. M. della Misericordia, 15, 33100 Udine, Italy; 2 Anesthesia and Intensive Care, Department of Emergency, Azienda per l’ Assistenza Sanitaria n° 3 Alto Friuli-Collinare-Medio Friuli, Tolmezzo, Italy; 3 Anesthesia and Intensive Care Clinic, Department of Anesthesia and Intensive Care Medicine, Academic Hospital “S. Maria della Misericordia”, Udine, Italy; 4 Full Professor of Anaesthesiology of the Department of Medical Area, University of Udine, Udine, Italy

**Keywords:** Rocuronium, Neostigmine, Reversal, Recovery time, Liver transplantation

## Abstract

**Background:**

Rapid neuromuscular block reversal at the end of major abdominal surgery is recommended to avoid any postoperative residual block. To date, no study has evaluated sugammadex performance after rocuronium administration in patients undergoing liver transplantation.

This is a randomized controlled trial with the primary objective of assessing the neuromuscular transmission recovery time obtained with sugammadex versus neostigmine after rocuronium induced neuromuscular blockade in patients undergoing orthotopic liver transplantation.

**Methods:**

The TOF-Watch SX®, calibrated and linked to a portable computer equipped with TOF-Watch SX Monitor Software®, was used to monitor and record intraoperative neuromuscular block maintained with a continuous infusion of rocuronium. Anaesthetic management was standardized as per our institution’s internal protocol. At the end of surgery, neuromuscular moderate block reversal was obtained by administration of 2 mg/kg of sugammadex or 50 mcg/kg of neostigmine (plus 10 mcg/kg of atropine).

**Results:**

Data from 41 patients undergoing liver transplantation were analysed. In this population, recovery from neuromuscular block was faster following sugammadex administration than neostigmine administration, with mean times±SD of 9.4 ± 4.6 min and 34.6 ± 24.9 min, respectively (*p* < 0.0001).

**Conclusion:**

Sugammadex is able to reverse neuromuscular block maintained by rocuronium continuous infusion in patients undergoing liver transplantation. The mean reversal time obtained with sugammadex was significantly faster than that for neostigmine. It is important to note that the sugammadex recovery time in this population was found to be considerably longer than in other surgical settings, and should be considered in clinical practice.

**Trial registration:**

ClinicalTrials.govNCT02697929 (registered 3rd March 2016).

## Background

Myoresolution plays a crucial role in laparotomic and laparoscopic general surgery, including orthotopic liver transplantation (OLT), where a deep level of neuromuscular block (NMB) has been shown to provide better surgical conditions [1, 2]. Deep NMB, defined as ≤2 responses after post-tetanic stimulation (or ‘post-tetanic count’, PTC), requires higher doses of a neuromuscular blocking drug (NMBD), with a consequent higher risk of longer and unpredictable recovery times regardless of the agent used [3–5]. The use of NMBD at high dosages to achieve deep NMB may also be associated with increased postoperative residual curarization (PORC) that leads to pulmonary complications and may even hinder successful extubation [6–8]. Both the administration of NMBD reversal agents at the end of surgery and the use of neuromuscular transmission (NMT) monitoring throughout the surgical procedure are key factors to counteract the adverse outcomes related to impaired neuromuscular transmission after extubation [9–12].

International guidelines and expert opinions strongly recommend NMT monitoring to optimize the dosage and timing of both NMBD and reversal agent administration, with the upshot of enabling early and safe extubation. This is also strongly encouraged in OLT settings, due to it being able to affect patient outcome directly as well as being a cost-effective practice [13–18].

The pharmacokinetics and pharmacodynamics of rocuronium bromide may be altered in patients with impaired liver function, resulting in a longer elimination half-life, a slower recovery from NMBD and unpredictable behaviour for instance about onset time [19, 20].

The safety and speed of action of sugammadex has been investigated and validated in different settings, including patients with liver disease undergoing liver surgery [21–23]. Fujita and colleagues demonstrated that sugammadex can be effective for the reversal of NMBD after low dose rocuronium continuous infusion in patients with liver disorders undergoing liver resection surgery, with no differences between cirrhotic and non-cirrhotic patients, as supported by comparable sugammadex recovery times between the two groups of patients [24]. Similar results were recently obtained by Abdulatif and colleagues who recorded a mean recovery time of 3.1 min for sugammadex given at a dose of 2 mg/kg for moderate block reversal in a cohort of patients with chronic hepatitis C liver cirrhosis undergoing liver resection surgery for neoplastic lesions [25].

However, patients with end-stage liver disease undergoing OLT may present some features that make the pharmacodynamics of rocuronium even more unpredictable; such features may include: extensive blood loss; need for urgent surgery; long length of surgery; extensive fluid shifts; large haemodynamic changes with consequent variable organ perfusion (including emunctory organs).

To the best of our knowledge, no study has evaluated sugammadex use after the continuous infusion of rocuronium during OLT. The primary objective of this study was to measure the time interval from the administration of the NMBD reversal agent (sugammadex or neostigmine) to a train-of-four ratio (TOF_R_) ≥ 0.9 (i.e., the recovery time) in patients who had undergone OLT with intraoperative continuous rocuronium infusion. The secondary objective was to determine possible relationships between the recovery times of the two drugs with pre-, intra- or postoperative variables.

## Methods

This is a single centre, non-blinded, randomized controlled trial approved by our Institution’s Ethics Committee “Comitato Etico Unico Regionale-CEUR” of Academic Hospital “*S. Maria* della Misericordia” (n° 2016-O-015-ASUIUD) and registered at ClinicalTrials.gov (NCT02697929). This manuscript adheres to the applicable CONSORT guidelines and the study protocol conformed to the Declaration of Helsinki ethical guidelines.

After Ethics Committee approval, we evaluated consecutive cases of OLT performed at the Academic Hospital “*S. Maria* della Misericordia”, Udine, Italy. Written informed consent was obtained before commencing OLT; patients were then randomly allocated to the sugammadex or neostigmine group using an online computer-generated table. All clinical data were collected in an Excel spreadsheet (Microsoft Excel for Mac, Version 14.0.0, Microsoft Corporation, USA). Inclusion criteria were as follows:

- Age > 18 years
- Neuromuscular transmission data acquired with TOF-Watch SX Monitor Software® (Organon, Dublin, Ireland. Version 1.2), from anaesthesia induction until extubation

Exclusion criteria were as follows:

- American Society of Anesthesiologists (ASA) status > 3
- Neuromuscular disease
- NMBA other than rocuronium bromide used
- Body mass index (BMI) < 18 kg/m^2^ or > 40 kg/m^2^
- Pre-operative impaired renal function, defined as an estimated glomerular filtration rate < 30 ml/min/1.73 m^2^
- Incorrect dosage of reversal (50 mcg/kg for neostigmine and 2 mg/kg for sugammadex)
- An inner body temperature < 35 °C or thenar temperature < 32 °C at reversal administration
- Haemodynamic instability defined as norepinephrine dosage > 0.1 mcg.kg^− 1^.min^− 1^ and/or dobutamine > 3 mcg.kg^− 1^.min^− 1^ and/or epinephrine > 0.1 mcg.kg^− 1^.min^− 1^ at the time of reversal administration and/or mean arterial pressure < 60 mmHg and/or HR > 100 bpm
- Acidosis defined as an arterial pH < 7.30 at the time of reversal administration

The last three exclusion criteria were also applied to determine whether extubation at the end of surgery could be attempted in the operating room in cases in which no graft dysfunction was present.

The pre-operative data collected regarded: sex; age; weight; body mass index (BMI); liver disease; model for end-stage liver disease (MELD); liver function; and renal function. The intra-operative data collected regarded: duration of surgery; intra-operative blood losses; packet red cells (PRC); fresh frozen plasma (FFP) and salvage blood transfused; platelets (PLT) use; fibrinogen administration; net fluid balance; cold ischaemia time (CIT); warm ischaemia time (WIT); total dose of rocuronium administered; and total millilitres of crystalloids and colloids infused. Cardiac output, cardiac index and central temperature (values obtained from the pulmonary artery catheter) with thenar temperature at the time of reversal administration were also recorded. Any adverse events were reported in the anaesthesia sheet.

Anaesthesia was induced with propofol (1–1.5 mg/kg) and fentanyl (3–5 mcg/kg) or alfentanil (7–15 mcg/kg) after facial mask denitrogenation with F_I_O_2_ = 0.8. NMBD (rocuronium 0.6 mg.kg based on lean body weight) was administered at anaesthesia induction only after TOF Watch SX® calibration (Organon, Dublin, Ireland). Anaesthesia was maintained with sevoflurane (End Tidal [ET%] targeted to keep the bispectral index in the 40–60 range) and the continuous infusion of remifentanil (0.05–0.3 mcg.kg^− 1^.min^− 1^), while neuromuscular block was maintained by continuous intravenous infusion of rocuronium bromide (Esmeron® 50 mg/5 mL, MSD Italia S.r.l., Roma) (0.3–0.6 mg.kg^− 1^.h^− 1^) to keep T_1_ < 1 0%. Haemodynamic monitoring, using a pulmonary artery catheter (CCOmbo catheter 777HF8; Edwards Lifescience, Irvine, California, USA), aimed to optimize indexed oxygen delivery (DOI_2_ > 600 ml.min^− 1^.m^2^) for the first 6 h postoperatively. All OLT were performed with the same surgical *equipe* dedicated to solid organ transplant surgery.

As per our routine practice for NMT monitoring with TOF-Watch, two electrodes were placed over the left ulnar nerve at the wrist and the acceleration transducer put on the thumb, together with a hand adapter that immobilized the other fingers. Data were collected using a dedicated computer and TOF-Watch SX Monitor Software®, which registered the response to ulnar nerve stimulation every 15 s. After anaesthesia induction, but before rocuronium administration, the TOF-Watch SX was calibrated. Once calibration was complete, the rocuronium bolus for tracheal intubation was administered and continuous infusion started.

At the end of surgery, reversal agents – sugammadex 2 mg/kg based on actual body weight (Bridion® 100 mg/mL, MSD Rome, Italy) or neostigmine 50 mcg/kg based on adjusted body weight plus 10 mcg/kg of atropine (Intrastigmina®, 0.5 mg/mL, Lusofarmaco S.p.a., Rozzano, Italy) – were administered (according to randomization) after the appearance of three consecutive T_2_ twitches (the so called moderate neuromuscular block; T2) detected by TOF Watch SX®. Recovery time was defined as the time interval from the administration of the reversal agent to the achievement of 3 consecutive measurements of TOF_R_ ≥ 0.9.

Our secondary objective was to analyse the main possible correlations between factors that may have influenced sugammadex and neostigmine recovery time: BMI; MELD; pre-operative and postoperative liver and renal function; surgical procedure length; blood loss; intraoperative fluid balance; cold and warm ischaemia time; total amount of NMBA delivered; millilitres of crystalloid and colloid infused; CO and CI at the time of reversal administration.

### Statistical analysis

Illman and colleagues found a mean difference of 11.6 min between sugammadex and neostigmine recovery times, administered when two twitches were detectable and reversal considered as having occurred when TOF_R_ > 90% [26]. Considering a 1:1 treatment ratio and an expected 5-min reduction in the sugammadex group, with an alpha level of 0.05 and a power (1-ß) of 90%, the calculated sample size was 16 subjects for each group. Taking into consideration a potential dropout of 20% and to increase the statistical significance, we decided to enrol at least 20 subjects per group.

Descriptive statistics (mean and standard deviation for quantitative variables, and absolute and relative frequencies for qualitative variables) were calculated for each group. To test for a difference in the recovery times between the two groups with respect to the primary objective, we implemented a two-sided unpaired *t* test as well as an F-test to compare variances and to test whether the *t* test assumptions were met. The same test was applied to all remaining data. The alpha level of statistical significance for all applied tests was 0.05. No imputation of missing data was used in the analysis. Finally, to detect possible relationships between the recovery times of the two drugs with other variables, we performed both the Spearman rank and the Pearson correlation tests, since differences, or a lack thereof, could provide additional information.

GraphPad Prism version 6.01 (GraphPad Software, California, USA) was used for the final statistical analysis.

## Results

**Fig. 1:**
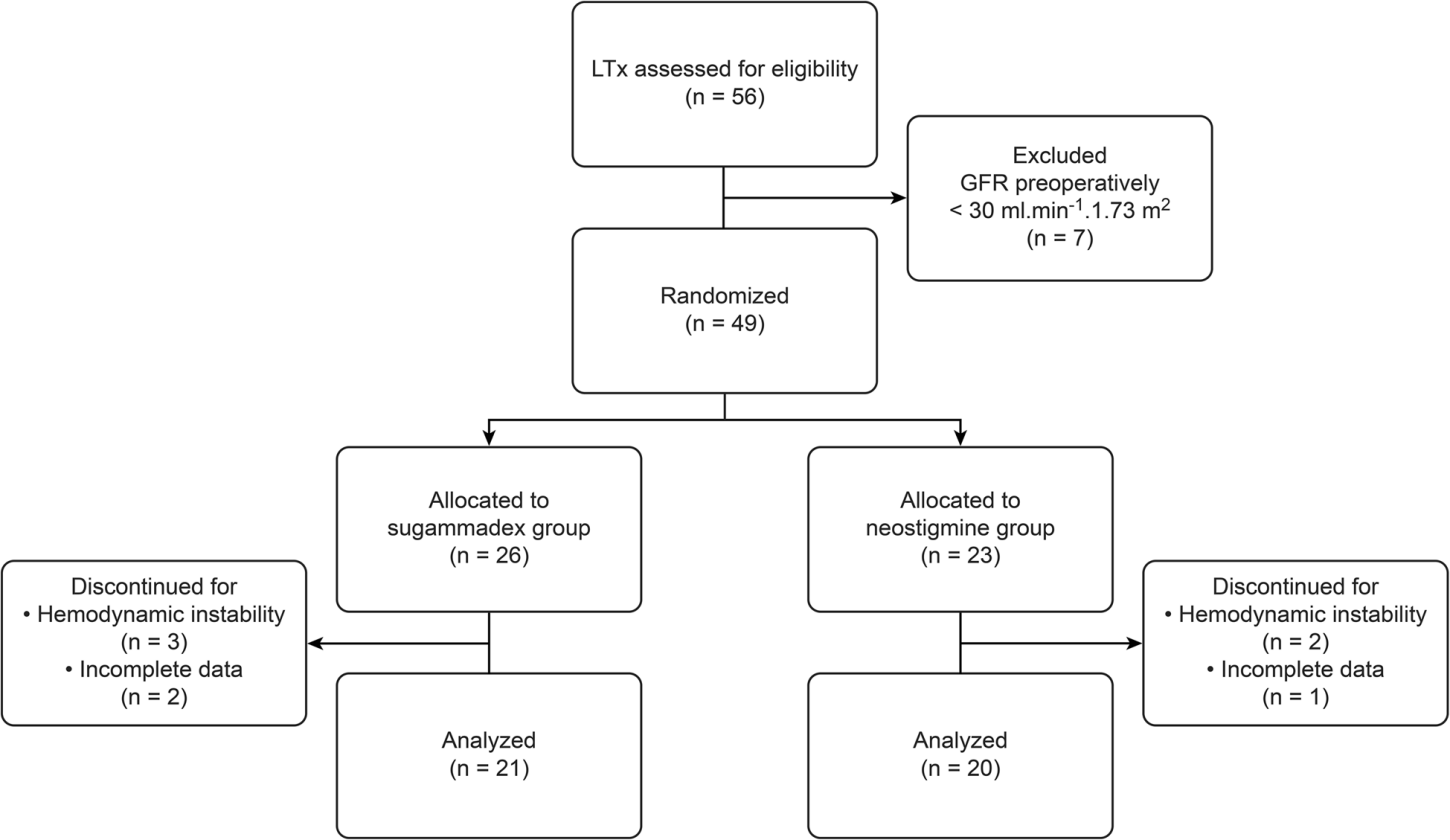
Study flow-chart according to CONSORT. *LTx*, orthotopic liver transplantation; *GFR*, glomerular filtration rate

A total of 41 patients were enrolled onto the study: 21 were treated with sugammadex and 20 with neostigmine, as shown in the study flowchart (*Fig.**1*). Baseline characteristics and end-stage liver disease aetiology were comparable between the sugammadex and neostigmine groups (*Table**1*). Furthermore, there were no statistically significant differences in pre-, intra- or postoperative values regarding haemodynamics, liver and renal function (*Table**1**and**2*).

**Table 1:** Demographics, biometrics, pre and postoperative liver and renal function data. Values are expressed as the mean ± SD unless otherwise indicated. Postoperative data refers to laboratory tests conducted when the patient was admitted into the ICU after LTx.

	Sugammadex *n* = 21	Neostigmine *n* = 20	
Gender			
*female, n and (%)*	7 (33)	8 (40)	
*male, n and (%)*	14 (67)	12 (60)	
Age (years)	54.1 ± 9.8	54.1 ± 10.4	
Weight (kg)	73.9 ± 14.2	73.1 ± 14.7	
Height (m)	1.7 ± 0.1	1.7 ± 0.1	
BMI (kg/m^2^)	25.2 ± 4.2	25.3 ± 3.9	
Liver disease:			
*HBV-related cirrhosis ± HCC, n and (%)*	2 (9.5)	0 (0)	
*HCV-related cirrhosis ± HCC, n and (%)*	9 (42.8)	6 (30)	
*Alcohol-related cirrhosis ± HCC, n and (%)*	6 (28.5)	6 (30)	
*Alchohol + HBV-related cirrhosis, n and (%)*	1 (4.7)	0 (0)	
*Alchohol + HCV-related cirrhosis, n and (%)*	0 (0)	4 (20)	
*HIV coexists, n and (%)*	2 (9.5)	2 (9.5)	
*Other disease, n and (%)*	3 (14.2)	4 (19)	
Pre-operative liver and renal function			*p*
*MELD*	16.8 ± 7.3	21.4 ± 9.1	0.1
*AST (IU/l)*	67.4 ± 39.1	122.8 ± 62	0.15
*ALT (IU/l)*	48.6 ± 41.1	89.7 ± 168.7	0.30
*LDH pre-op (IU/l)*	485.3 ± 191.5	457.8 ± 368.4	0.93
*TBil (mg/dl)*	6.7 ± 10.2	11.5 ± 13.4	0.45
*DBil (mg/dl)*	3.8 ± 6.1	6.1 ± 8.2	0.6
*GGT (IU/l)*	155 ± 340	75 ± 74	0.34
*Albumin (mg/dl)*	29.9 ± 6.2	31 ± 6.4	0.27
*ClCr (ml/min)*	86.5 ± 34.9	80.7 ± 36.8	0.61
Postoperative liver and renal function			
*AST (IU/ml)*	1246 ± 1060	1688 ± 1508	0.29
*ALT (IU/m)*	654 ± 348	1346 ± 1594	0.07
*LDH (IU/l)*	2986 ± 1699	3957 ± 5804	0.49
*TBil (mg/dl)*	4.6 ± 2.5	6.9 ± 4.6	0.25
*DBil (mg/dl)*	3.2 ± 2	4 ± 3.2	0.65
*GGT (IU/l)*	58 ± 47	77 ± 56	0.27
*Albumin (mg/dl)*	19.8 ± 8	21.3 ± 6	0.82
*ClCr (ml/min)*	86.2 ± 30.7	72.2 ± 29.1	0.14

Abbreviations: *BMI* body mass index; *MELD* Model for End-Stage Liver Disease; *AST* aspartate aminotransferase; *ALT* alanine aminotransferase; *LDH lactate dehydrogenase; TBil* total bilirubin; *DBil* direct bilirubin; *GGT* gamma glutamyl-transferase; *ClCr* estimated creatinine clearance (Cockroft-Gault formula used)

**Table 2:** Intraoperative data. Cardiac output and index were evaluated at reversal administration. Values are expressed as the mean ± SD.

	Sugammadex (n = 21)	Neostigmine (n = 20)	*p*
*Duration of surgery (min)*	402 ± 54	378 ± 102	0.41
*Intra-op blood losses (ml)*	7278 ± 6346	6039 ± 5608	0.56
*PRC (ml)*	1700 ± 1325	2407 ± 2475	0.28
*FFP transfused (ml)*	2981 ± 2492	2933 ± 3207	0.9
*Salvaged blood transfused (ml)*	1825 ± 1750	1650 ± 2147	0.81
*Fluid balance (ml)*	3374 ± 2512	1612 ± 1915	0.07
*CIT (h)*	8.3 ± 1.9	8.8 ± 2.1	0.73
*WIT (min)*	46.6 ± 16.5	39 ± 14.8	0.12
*Total crystalloid infusion (ml)*	8585 ± 3224	6582 ± 3095	0.06
*Total albumin 4% infusion (ml)*	2612 ± 1314	1982 ± 1199	0.14
*Cardiac Output (l/min)*	8.6 ± 2.2	8.8 ± 2.8	0.80
*Cardiac Index (l/min/m*^*2*^*)*	4.8 ± 1.2	4.8 ± 1.7	0.91

Abbreviations: *PRC* packet red blood cell; *PLT* platelets; *FFP* fresh frozen plasma; *CIT* cold ischaemia time; *WIT* warm ischaemia time

The rocuronium onset times were similar between the two groups at 195 ± 124 and 250 ± 123 s for the sugammadex and neostigmine groups, respectively (*p* = 0.20). The total dose of rocuronium administered was 217 ± 61 mg and 199 ± 74 mg (*p* = 0.41) for the sugammadex and neostigmine groups, respectively. The total amount of reversal administered was 147 ± 25.5 mg and 3.7 ± 0.6 mg, respectively.

The mean core and thenar site temperatures of patients treated with sugammadex, recorded at reversal administration, were 37 ± 0.8 °C and 35.2 ± 1.3 °C, respectively; whereas they were 36.6 ± 0.9 °C and 35.2 ± 1.4 °C in the neostigmine group (core *p* = 0.29, thenar site *p* = 0.92).

**Fig. 2:**
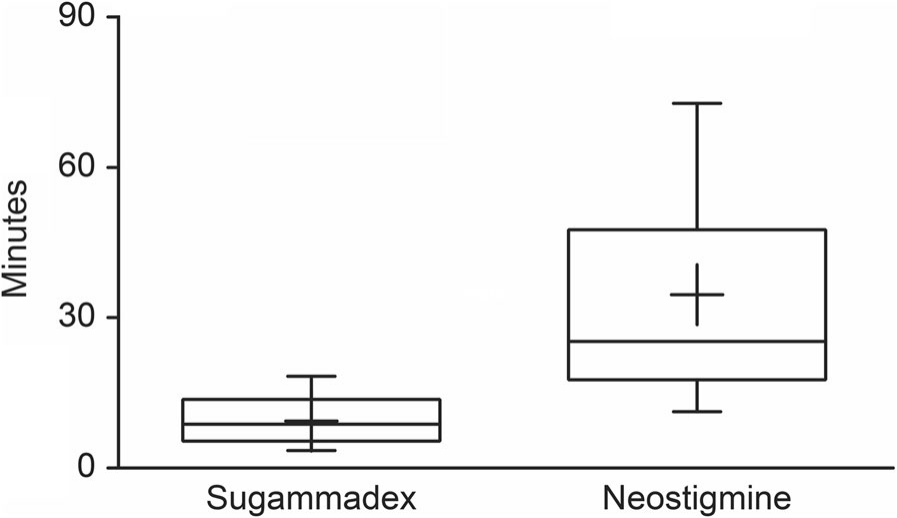
Recovery time for sugammadex and neostigmine. Mean value was 9.4 vs. 34.6 minutes for sugammadex and neostigmine respectively (*p* < 0.0001)

The mean time from ceasing rocuronium infusion to T2 was 57 ± 32 and 58 ± 25 min in the sugammadex and neostigmine groups, respectively (*p* = 0.95). Mean recovery times were significantly faster in patients treated with sugammadex than neostigmine: 9.4 ± 4.6 min vs. 34.6 ± 24.9 min, respectively (*p* < 0.0001), as shown in *Fig.**2*. Seven patients out of 21 (33%) in the sugammadex group required more than 10 min to achieve a TOF_R_ > 0.9. One patient in the neostigmine group was an outlier with a recovery time > 100 min and as such was not included in the mean recovery time calculation (Fig. 2).

In the sugammadex group, 4 patients were extubated at the end of surgery in the operating room (19%); this occurred in 2 patients in the neostigmine group (10%, *p* = 0.41). No difference was observed in the time from ICU admission to extubation (245 ± 22 min vs. 265 ± 62 min in the sugammadex and neostigmine groups, respectively, *p* = 0.44).

Two patients (9.5%) in the sugammadex group and 1 patient (5%) in the neostigmine group underwent re-operation within 24 h of extubation. In all cases, intra-abdominal haemorrhage was responsible for the redo surgery, and rapid sequence induction with succinylcholine was performed before administering cisatracurium.

The results of the coagulation tests performed before and at the end of surgery were as follows: neostigmine group: pre- vs. post operative INR: 2 ± 0.7 vs. 1.5 ± 0.4, respectively (*p* = 0.01); pre- vs. post operative aPTT ratio: 1.5 ± 0.5 vs. 1.2 ± 0.2, respectively (*p* = 0.04). The same tests relative to the sugammadex group were as follows: pre- vs. post operative INR: 1.6 ± 0.7 and 1.5 ± 0.5, respectively (*p* = 0.71); pre- vs. post operative aPTT ratio: 1.3 ± 0.4 vs. 1.3 ± 0.2, respectively (*p* = 0.43).

We also investigated the existence of correlations between recovery time and BMI, MELD, duration of surgery, WIT, CIT, PLT, fibrinogen, fluid balance, amount of intravenous fluids administered, liver function, creatinine clearance pre or post-surgery and total amount of NMBDs administered. No evidence for any strong correlations were found using Pearson and Spearman tests (*Table**3*). Only a positive trend was highlighted that might denote a possible moderate correlation between sugammadex recovery time and: AST post OLT (r = 0.611, *p* = 0.003); ALT post OLT (r = 0.50, *p* = 0.02); and amount of colloids (r = 0.50, p = 0.02). In the neostigmine group, only length of surgery showed a possible moderate correlation with recovery time (r = 0.57, *p* = 0.009). No adverse events were reported for either group.

**Table 3:** Correlations between recovery time and pre, intra and postoperative values. “Pre” refers to pre-operative while “post” to postoperative value.

	SUGAMMADEX				NEOSTIGMINE			
	Pearson	***p***	Spearman	***p***	Pearson	***p***	Spearman	***p***
**BMI**	−0.304	0.180	− 0.342	0.129	−0.119	0.618	0.105	0.659
**MELD**	0.195	0.454	0.046	0.862	− 0.416	0.076	−0.399	0.091
**AST pre**	0.275	0.253	0.341	0.152	−0.191	0.419	−0.146	0.537
**AST post**	0.611	0.003	0.569	0.007	0.088	0.710	0.330	0.154
**ALT pre**	0.1633	0.504	0.329	0.167	−0.165	0.486	−0.267	0.253
**ALT post**	0.4960	0.022	0.345	0.125	0.071	0.764	0.257	0.273
**GGT pre**	−0.061	0.815	0.335	0.186	0.429	0.075	0.100	0.692
**GGT post**	−0.242	0.317	0.037	0.878	−0.052	0.831	−0.012	0.960
**ClCr pre**	0.378	0.090	0.296	0.191	−0.124	0.601	−0.021	0.929
**ClCr post**	−0.232	0.311	−0.176	0.445	0.019	0.936	0.068	0.777
**Length surg**	0.293	0.198	0.357	0.113	0.570	0.009	0.341	0.142
**Blood loss**	0.423	0.063	0.355	0.123	0.239	0.355	0.169	0.513
**Fluid balance**	0.293	0.270	0.209	0.436	−0.131	0.630	−0.198	0.454
**CIT**	−0.151	0.515	−0.152	0.511	−0.084	0.726	0.083	0.726
**WIT**	0.461	0.036	0.372	0.097	−0.054	0.821	−0.049	0.837
**Tot NMBA**	0.271	0.235	0.182	0.430	0.135	0.582	0.268	0.267
**Crystalloids**	0.093	0.697	0.045	0.852	0.199	0.443	0.202	0.434
**Colloids**	0.502	0.024	0.271	0.248	0.041	0.875	0.091	0.727
**CO**	0.449	0.047	0.363	0.115	−0.051	0.845	0.015	0.957
**CI**	0.404	0.086	0.349	0.143	−0.062	0.813	0.091	0.727

Abbreviations: *BMI* body mass index; *MELD* Model for End-Stage Liver Disease; *CIT* cold ischaemia time; *WIT* warm ischaemia time; *ClCr* clearance creatinine (Cockroft Gault); *CO* cardiac output; *CI* cardiac index; *NMBA* neuromuscular blocking agent; *AST* alanine aspartate transferase; alanine amino transferase; *GGT* gamma glutamyl transferase

## Discussion

The present study constitutes the first randomized controlled trial to evaluate sugammadex use following a rocuronium bolus and continuous infusion in the OLT setting. Our main finding is that, as was expected, sugammadex results in a considerably shorter recovery time than neostigmine, although the absolute value was longer than expected, considering previously reported data in the literature regarding other (non OLT) operative settings. The mean recovery time for sugammadex in our population was 9.4 min, which is longer than that reported in other studies after sevoflurane anaesthesia [15], with 33% of patients requiring more than 10 min to achieve a TOF_R_ > 0.9. This should be taken into account in clinical practice when waking patients up at the end of transplantation. However, sugammadex retained its well-known advantage as it regards recovery time over neostigmine, which had a mean recovery time of 34.6 min. Of important note is the fact that 19% of the patients in the neostigmine group required more than 60 min to recover from NMB, with a maximum value that exceeded 100 min.

Previous studies using rocuronium-sugammadex in patients affected by liver disease undergoing hepatic resection have demonstrated the well-known advantages of sugammadex over neostigmine in the above-mentioned setting [24, 25]. However, OLT differs greatly from general surgery or liver resection surgery; for instance, blood loss and duration of surgery may be significantly greater in OLT. Furthermore, the severity of the liver disease can play a role: the patients enrolled onto our study were more critical than those enrolled by Fujita and colleagues [24] and Abdutalif and colleagues [25] given their end-stage liver disease (see the AST and ALT, bilirubin, serum albumin and gamma glutamyl transferase values for example). In addition, in the study by Fujita and colleagues, rocuronium continuous infusion was targeted at obtaining T1 at TOF-stimulation, a lower degree of NMB compared with our study, in which deep NMB was achieved. These findings probably explain the lower total dose of rocuronium infused in the above-cited studies compared with our study.

Moreover, some evidence exists in the literature, considering various operative settings, to suggest that higher total doses of rocuronium may be related to longer recovery times; for example, Llaurado and colleagues described a relationship between total rocuronium dose and recovery time in a cohort of obese patients following sugammadex administration [27]. Abdulatif and colleagues demonstrated the safe use and efficacy of sugammadex in the specific setting of cirrhotic patients with a mean recovery time of 3.1 min [25]. The higher rocuronium dosage used in the present study compared with that by Abdulatif et al. could, in part, explain our longer recovery times. It should also be noted, however, that the total duration of surgery required in our setting was significantly longer than in the cases considered by Abdulatif et al.; we administered rocuronium via continuous infusion at a fixed dose (in accordance with the study protocol), a modality that could have overexposed the patient (for instance in the anhepatic phase) to the NMBD.

In the literature, an experimental study has investigated the possibility of interactions occurring between sugammadex and other drugs, and indeed it has been found that flucloxacillin, fusidic acid and tormifene have the potential to exert a displacement interaction with sugammadex [28]. An in vitro study has also demonstrated possible interferences between corticosteroids and sugammadex action [29], whereas, Rezonja and colleagues, published an in vivo randomized controlled trial in which they demonstrated that dexamethasone does not alter sugammadex recovery time [30]. The opposite was later concluded by Saleh and colleagues, who, evaluating dexamethasone use for the prevention of post-operative nausea and vomiting in children undergoing strabismus surgery, demonstrated a delayed reversal of rocuronium-induced NMB by sugammadex [31]. To make the picture even more complex, it was recently demonstrated by Ozer and colleagues that sugammadex recovery times are reduced when administered in conjunction with steroids, especially desamethasone [32].

All the patients in our study received a high intraoperative dose (3.5–5 mg/kg) of methylprednisolone hemisuccinate as immunosuppressant just before hepatic vascular unclamping. In an experimental study on rats, Saleh and colleagues evaluated the effect of very high (non clinical) steroid dosages on the resulting behaviour of sugammadex on rocuronium, but, once again, no significant affect was detected [31]. Human studies into the potential interference between corticosteroids and sugammadex action are still lacking.

Kandemir and colleagues, in their animal study, found that when methylprednisolone was used in combination with remifentanil, sugammadex action was prolonged due to a synergistic effect [33]. This result contrasts with that reported by Zwiers and colleagues, in which these two drugs were used individually and no alterations in the clinical effect of sugammadex were reported [28]. However, in the different context of OLT, the pharmacokinetics of remifentanil are best described by a two-compartment model that takes into account a central and a peripheral volume of distribution. In addition, the functional status of the liver did not significantly affect the pharmacokinetics of remifentanil, although body weight also influences the volumes of distribution with implications for the pharmacokinetic behaviour of remifentanil [34].

These findings are very intriguing and require further research since the volume of distribution after OLT may be deeply altered – often increased – and could, therefore, lead to the redistribution of remifentanil and, consequently, to the possible interaction between remifentanil-methylprednisolone and sugammadex function following liver reperfusion. However, this is only a speculative proposal to explain the longer recovery times of sugammadex in OLT.

To understand the unexpected relatively long recovery time of sugammadex in more detail, we performed Pearson and Spearman tests to explore the possible correlations between peri-operative variables and recovery time, but no statistically significant strong correlations were found (Table 3). However, postoperative liver AST and ALT, and the amount of intra-operative colloids were found to show a positive trend with shorter sugammadex recovery times, denoting a possible moderate correlation. Intra-operative colloids may influence cardiac output. Thus, higher amounts of colloid administration could result in a faster redistribution of rocuronium from the peripheral compartment into the central one, where it is then encapsulated by sugammadex and eliminated by the kidney. It may also be possible that graft function influences sugammadex performance, since rocuronium is mostly eliminated by liver uptake, thus contributing to the elimination of NMB by adding to the action of sugammadex. However, the sample size of this study was not calculated with the view of investigating these potential interactions with sugammadex, so further randomized controlled trials of adequate numerosity will be required to understand these possibilities any further.

The pharmacokinetics of rocuronium are highly variable in cirrhotic patients, with some authors noting longer onset and offset times [35–37]. Given this premise, a physician might argue against its use in patients undergoing OLT, in favour of a non-organ-specific metabolized NMB, such as cisatracurium, with a more favourable metabolism [38]. However, the possibility of rapidly reversing rocuronium activity with sugammadex offers advantages in the context of fast track surgery in general and in OLT in particular, providing anaesthetists with the possibility of extubating the patient in the operating room or shortly after intensive care admission. There is also evidence that early extubation after OLT improves patient outcome and saves costs [17, 18]. However this fast track approach needs to fulfil some important criteria, such as haemodynamic and respiratory stability, no expected graft dysfunction, no large intraoperative blood loss, normal pH, normothermia, uncomplicated surgery, and good teamwork between surgeons, anaesthesiologists and intensivists [39]. Obviously, complete recovery from NMB to avoid respiratory failure due to PORC is mandatory and rocuronium-sugammadex use provides a valid option, especially because extubation failure after surgery and the need for reintubation represent an important mortality risk factor [40].

A possible negative consequence of sugammadex use may become apparent in the case that urgent surgery is required within 24 h of transplant as some residual reversal activity may still be present due to the continual circulation of sugammadex in the blood. In this case, rapid sequence induction is unavoidable, necessitating the use of succynilcholine with its well-known side-effects .

An increased risk of bleeding after sugammadex administration has been reported by De Kam et al. [41] They described an increase in INR and the aPTT ratio time after sugammadex administration, albeit in the absence of any clinical impact. More recent evidence supports the older findings by De Kam [42, 43]. Our results did not reveal any increases in coagulation laboratory test values, confirming no clinically significant augmented bleeding after sugammadex has been administered.

In light of the results of our study, it may be advisable to administer sugammadex with a reasonable margin of time (i.e., 15 min) before the actual extubation of the OLT recipient patient.

A limitation to our study should also be noted; all the OLT patients included in the study were characterised by haemodynamic stability, so our findings can’t be extended to patients with poor haemodynamic conditions.

Anaesthetic management can be an important source of bias during OLT, but anaesthetic conduct in our study adhered very strictly to an internal protocol applied to all OLT. As a consequence, anaesthetic variability was minimal as the protocol regarded all aspects not related to NMB management. This ‘standardised’ anaesthetic practice in OLT translated into the lack of any statistically significant differences in intra-operative variables (such as bleeding, fluids or transfusions) between the sugammadex and neostigmine group. Additionally, all OLT were performed with the same surgical team dedicated to solid organ transplant surgery, further decreasing the potential for treatment bias, such as different surgery durations times.

## Conclusion

The performance of sugammadex was superior to that of neostigmine in terms of recovery time in the OLT setting. Sugammadex offers anaesthetists an efficacious and safe reversal option that can facilitate a fast track recovery protocol including more frequent extubation in the operating room that may lead to improved outcomes. However, anaesthetists must bear in mind the need for longer sugammadex recovery times compared with other surgical settings and be aware that NMT monitoring is mandatory.

Further randomized clinical trials are needed to further characterize sugammadex in the setting of liver transplant, including its longer recovery time versus other surgical populations, and the potential role of steroids on its clinical effect.

## Data Availability

The datasets used and/or analysed during the current study are available from the corresponding author on reasonable request.

## Abbreviations

OLT: Orthotopic liver transplantation
NMB: Neuromuscular block
NMBD: Neuromuscular blocking drug
PTC: Post tetanic count
PORC: Post operative residual curarization
NMT: Neuromuscular transmission
TOF_R_: Train of four ratio
BMI: Body mass index
MELD: Model for end stage liver disease
PRC: Pure red cell
FFP: Fresh frozen plasma
PLT: Platelets
CIT: Cold ischemia time
WIT: Warm ischemia time
CO: Cardiac output
CI: Cardiac index
